# Anti-Allergic Properties of Propolis: Evidence From Preclinical and Clinical Studies

**DOI:** 10.3389/fphar.2021.785371

**Published:** 2022-01-21

**Authors:** Kong Yen Liew, Nurain Irdayani Kamise, Hui Ming Ong, Poi Yi Aw Yong, Fahmida Islam, Ji Wei Tan, Chau Ling Tham

**Affiliations:** ^1^ Department of Biomedical Sciences, Faculty of Medicine and Health Sciences, Universiti Putra Malaysia, Serdang, Malaysia; ^2^ School of Science, Monash University Malaysia, Subang Jaya, Malaysia

**Keywords:** propolis, allergy, asthma, allergic rhinitis, atopic dermatitis, eczema, mast cell, basophil

## Abstract

Allergic diseases are a global health burden with increasing prevalence. Side effects of available medications (antihistamines and steroids), lack of patients’ perceived effectiveness and high cost of biologic therapies (omalizumab) are challenges to the clinical management of allergic diseases. As allergy symptoms persist for a long time, complementary and alternative medicine (CAM) such as propolis may be considered a potential prophylactic or therapeutic option to avoid long-term medication use. Propolis is a natural resinous substance produced by bees. Although propolis is well known to possess antioxidant, antimicrobial, and anticancer properties, its anti-allergic potential is not fully explored. Several preclinical studies demonstrated the therapeutic effects of propolis extracts against allergic inflammation, asthma, allergic rhinitis, atopic dermatitis, and food allergy, which may be partly attributed to their inhibitory effects on the activation of mast cells and basophils. Clinically, the consumption of propolis as a supplement or an adjunct therapy is safe and attenuates various pathological conditions in asthma. Such an approach may be adopted for atopic dermatitis and allergic rhinitis. Although flavonoids (chrysin, kaempferol, galangin, and pinocembrin) and cinnamic acid derivatives (artepillin C and caffeic acid phenethyl ester) can contribute to the anti-allergic activities, they may not be present in all propolis samples due to variations in the chemical composition. Future studies should relate the anti-allergic activity of propolis with its chemical contents. This mini-review summarizes and discusses existing preclinical and clinical studies reporting the anti-allergic activities of propolis to provide insights into its potential applications in allergic diseases.

## Introduction

Allergic diseases are a global health problem affecting approximately 20–30% of the population in industrialized countries (Akdis, 2014). The prevalence of allergic diseases, such as allergic asthma, allergic rhinitis, atopic dermatitis, anaphylaxis, and food and drug allergies, is increasing worldwide, especially in low- and middle-income countries (Pawankar, 2014). Allergic diseases are complex and cause a broad spectrum of symptoms ranging from mild to severe and potentially life-threatening anaphylactic reactions (Kun and Li, 2016).

In allergic diseases, mast cells, basophils, and eosinophils are the principal effector cells that regulate allergic inflammation. The binding of allergen to immunoglobulin E (IgE) causes cross-linking of IgE receptors such as FcεRI and activates mast cells and basophils. This results in their degranulation and release of inflammatory mediators such as histamine, β-hexosaminidase, prostaglandins (PGs), cysteinyl leukotrienes (CysLTs), as well as pro-inflammatory cytokines and chemokines such as interleukin (IL)-4, IL-6, IL-8, IL-13, and tumour necrosis factor (TNF)-α (Stone et al., 2010). These mediators can cause rashes and itchy skin, sneezing, bronchoconstriction, and eosinophilic inflammation (Stone et al., 2010). Although antihistamines (loratadine and cetirizine) and anti-inflammatory drugs (inhaled and topical steroids) remain the mainstay of treatment for allergic diseases (Kruse and Vanijcharoenkarn, 2018), side effects such as nasal irritation and epistaxis, lack of patients’ perceived effectiveness and high cost of biologic therapies targeting IgE (omalizumab) are challenges to the clinical management of allergic diseases (Kruse and Vanijcharoenkarn, 2018; Baiardini et al., 2019). Since allergy symptoms can persist for a long time and severely impact the patients’ quality of life, complementary and alternative medicine (CAM) like propolis may be considered one of the prophylactic or therapeutic options for allergic diseases to avoid long-term medication use.

Propolis, also known as “bee glue,” is a natural resinous substance produced by bees to protect their hives and combat infections. It is rich in flavonoids, terpenoids, phenolics and their esters, with more than 500 chemical compounds identified in propolis (Huang et al., 2014). Propolis has been traditionally used to treat burns, ulcers, asthma, and diabetes (Kuropatnicki et al., 2013). Besides that, propolis has been demonstrated to exhibit excellent anti-inflammatory, antimicrobial, and anticancer properties, and these have been extensively reviewed (Pasupuleti et al., 2017; Anjum et al., 2019; Zulhendri et al., 2021). However, there are not many studies investigating the anti-allergic properties of propolis. Thus, the objective of this mini-review is to summarize and critically discuss existing studies reporting the anti-allergic activities of propolis in both preclinical and clinical studies to provide insights into its potential applications in allergic diseases.

## Preclinical Studies on the Effects of Propolis in Various Cellular and Animal Models of Allergic Diseases

### Allergen-Induced Allergic Inflammation

An *in vitro* study by Khosravi et al. (2018) evaluated the effects of the ethanol extract of propolis collected from the hives of *Apis mellifera* L. bees of southern Iran on cytokine production in murine lung epithelial cells TC-1 JHU-1 induced with a fungal allergen, *Aspergillus fumigatus* conidia (Khosravi et al., 2018) (Table 1A). Propolis ethanol extract treatment has been shown to suppress the production of IL-13 and IL-17 pro-inflammatory cytokines and increase IL-12 production. *A. fumigatus* can cause allergic inflammation in people suffering from allergic bronchopulmonary aspergillosis (ABPA), allergic Aspergillus sinusitis, and asthma. Thus, these findings suggest the potential therapeutic effect of propolis for allergic disorders.

**TABLE 1:** (A) Preclinical studies investigating the effects of propolis in different cellular and animal models of allergic diseases. (B) Clinical studies investigating the effects of propolis in different allergic diseases.

Type of allergic disease	Type of propolis	Experimental model; inducer	Treatment concentration/dose	Mode of treatment; route of administration	Experimental outcome	References
Allergen-induced allergic inflammation	*Apis mellifera* L. bees propolis ethanol extract (Iran)	*In vitro* (murine lung epithelial cells TC-1 JHU-1); *Aspergillus fumigatus* conidia	25 μg/ml	Co-treatment (6 or 12 h); NA	↓ IL-13 and IL-17	Khosravi et al. (2018)
					↑ IL-12	
	Brazilian green propolis ethanol extract	*Ex vivo* (peripheral leukocytes and peripheral blood mononuclear cells from 10 allergic patients); Cry j1	3, 10, 30 and 100 μg/ml	Co-treatment (6 days); NA	In Cry j1-induced peripheral leukocytes	Tani et al. (2010)
					↓ cysLTs	
					↓ histamine	
					In Cry j1-induced peripheral blood mononuclear cells	
					↓ IL-5 and IL-13	
Mast cell degranulation	Water extract (WEP) and ethanol extract (EEP) of propolis from China and Brazil	*In vitro* (RBL-2H3); Anti-DNP IgE sensitization followed by DNP-BSA challenge	0.01, 0.1 and 1% (WEP and EEP)	Pre-treatment (30 min, 3 h or 18 h); NA	WEP and EEP from China and Brazil	Nakamura et al. (2010)
					↓ β-hexosaminidase release	
	Brazilian green propolis ethanol extract	*Ex vivo* (rat peritoneal mast cells); antigen or compound 48/80	3, 10, 30 and 100 μg/ml	Pre-treatment (10 min); NA	↓ histamine release	Shinmei et al. (2004), Shinmei et al. (2009), Shinmei et al. (2010)
Basophil-mediated allergic inflammation	Brazilian green propolis powder	*In vivo* (BALB/c mice); Anti-DNP IgE sensitization followed by intradermal DNP_11_-OVA challenge	0.3 mg	Treatment on each of 3 alternate days before anti-DNP IgE sensitization; Intragastric	↓ ear skin thickness	Kashiwakura et al. (2021)
					↓ inflammation and leukocytes infiltration in ears	
					↓ Mcpt8 gene expression in ear tissues	
		*In vivo* (BALB/c mice); OVA	0.3 mg	Twice a week for 3 weeks from day 28; Oral	↓ diarrhoea occurrence	
					↓ clinical scores	
					↓ serum mMCP-1	
					↓ IL-4 and E-NPP3 gene expression in jejuna	
		*Ex vivo* (bone marrow-derived basophils from BALB/c mice); Anti-DNP IgE sensitization followed by DNP_23_-HSA challenge	1, 10 and 100 μg/ml	Pre-treatment (6 h) or co-treatment (24 h); NA	↓ phosphorylation of FcεRI signalling molecules (Lyn, Akt and Erk)	
					↓ IL-4, IL-6 and IL-13 production	
Asthma	Propolis water extract powder (Taiwan)	*In vivo* (6–8 weeks old female BALB/c mice); OVA	65 and 325 mg/kg	Daily for up to 9 weeks after second OVA sensitization; Oral	↓ serum OVA-specific IgE and IgG_1_	Sy et al. (2006)
					↑ serum OVA-specific IgG_2a_	
					↓ airway hyperresponsiveness	
					↓ IL-5 in BALF	
					↓ lung inflammation	
		*Ex vivo* (splenocytes from OVA-induced asthmatic mice); concanavalin A or OVA			In ConA-induced splenocytes	
					↑ IFN-γ	
					↓ IL-10	
					In OVA-induced splenocytes	
					↓ IL-6, IL-10 and IFN-γ	
	Propolis hydroalcoholic extract produced by *Scaptotrigona* aff. *postica* stingless bee (Brazil)	*In vivo* (2–3 months old female BALB/c mice); OVA	50 and 200 mg/kg	Daily for 2 weeks after second OVA sensitization; Oral	↓ total cell counts and polymorphonuclear cells in BALF	De Farias et al. (2014)
					↓ peribronchovascular inflammation and epithelial desquamation	
					↓ serum IFN-γ	
	Propolis aqueous and ethanol crude extract (Egypt)	*In vivo* (6 weeks old male albino CD1 mice); conalbumin	30 mg/kg	Daily for 18 days after second conalbumin sensitization; Intraperitoneal	↓ blood eosinophils and basophils	El-Aidy et al. (2015)
					↓ lung inflammation (peribronchial and perivascular inflammatory cell)	
	Standardized Brazilian green propolis extract (EPP-AF^®^ extract)	*In vivo* (6–8 weeks old female C57BL/6 mice); OVA	150 mg/kg	Daily for 17–22 days after second OVA sensitization; Oral	↓ pulmonary inflammation	Piñeros et al. (2020)
					↓ mucus production	
					↓ IL-5 and eosinophils in BALF	
					↓ IL-13 gene expression in lungs	
					↓ eosinophils and M2 macrophages in lungs	
					↑ PMN-MDSC and CD4^+^ Foxp3+ regulatory T cells in lungs	
Allergic rhinitis	Brazilian green propolis granular	*In vivo* (5 weeks old male BALB/c mice); OVA or histamine	200, 500 and 1,000 mg/kg	Daily for 4 weeks; Oral	Repeated administration of propolis (2–4 weeks)	Shinmei et al. (2009)
					↓ sneezing and nasal rubbing	
	Propolis ethanol extract (poplar propolis collected from honeybee colonies of *Apis mellifera caucasica*, Turkey)	*In vivo* (male Sprague-Dawley rats aged ≥6 weeks); OVA	200 mg/kg	Daily for 21 days after last OVA sensitization; Oral and intranasal	Oral propolis	Yasar et al. (2016)
					↓ ciliary loss, inflammation, vascular proliferation, increase in goblet cells and eosinophils in the nasal mucosa	
					↓ symptom scores (nasal irritation, sneezing and amount of nasal secretion) on days 1–4	
					Intranasal propolis	
					↓ vascular congestion, eosinophils and chondrocytes in the nasal mucosa	
					↓ symptom scores (nasal irritation, sneezing and amount of nasal secretion) on days 1, 3 and 4	
	Brazilian green propolis ethanol extract	*In vivo* (6 weeks old male Brown Norway rats); toluene 2,4-diisocyanate (TDI)	40 and 80 mg/kg	Daily for 3 weeks before TDI sensitization; Oral	↓ sneezing	Shaha et al. (2018)
					↓ nasal scores (watery rhinorrhea, swelling and redness)	
					↓ H1R mRNA levels in the nasal mucosa	
					↓ IL-4, IL-5 and IL-9 mRNA levels in the nasal mucosa	
		*In vitro* (HeLa); histamine or phorbol-12-myristate-13-acetate (PMA)	25, 50 and 75 μg/ml	Pre-treatment (3 h); NA	↓ H1R gene expression	
					↓ PKCδ phosphorylation at Tyr311	
		*In vitro* (RBL-2H3); ionomycin	25, 75 and 100 μg/ml	Pre-treatment (3 h); NA	↓ IL-9 gene expression	
Atopic dermatitis	Brazilian green propolis granular	*In vivo* (6–10 weeks old female ICR rats); compound 48/80 or histamine (intradermal injection)	200, 500 and 1,000 mg/kg	Daily for 4 weeks; Oral	In rats induced with compound 48/80	Shinmei et al. (2004)
					↓ scratching behaviour	
					↓ skin vascular permeability	
	Brazilian green propolis ethanol extract	*In vivo* (6–10 weeks old female ICR rats); compound 48/80 or histamine	0.3, 1 and 3 mg/site	Applied 60 min before compound 48/80 or histamine injection; Topical	In rats induced with compound 48/80	Shinmei et al. (2010)
					↓ scratching behaviour (0, 15, 30 and 60 min)	
					↓ skin vascular permeability	

Type of allergic disease	Type of propolis	Subjects (age, gender and number)	Treatment method and frequency	Experimental outcome	Anti-allergic properties (Y/N)	References
Asthma	Propolis aqueous extract (Propharma, Stenlose, Denmark) prepared from crude propolis collected from Denmark, China, Uruguay and Brazil	Individuals with mild to moderate asthma for the last 2–5 years	Propolis group: oral theophylline + propolis-containing sachet	↓ nocturnal attacks	Y	Khayyal et al. (2003)
		19–52 years; 36 male, 10 female	Placebo group: oral theophylline + placebo-containing sachet	↑ pulmonary ventilatory functions (FVC, FEV1, PEFR, FEF25-75)		
		Propolis (*n* = 24)	Once daily for 2 months	↓ serum TNF-α, IL-6, IL-10 and ICAM-1		
		Placebo (*n* = 22)		↑ serum IL-10		
				↓ serum PGE_2,_ PGF_2α_ and LTD_4_		
	Propolis cera tablet (Soren Tech Toos Company, Mashhad, Iran)	Individuals with newly diagnosed, previously untreated moderate asthma	Propolis group: 3 propolis tablets/day (75 mg propolis)	↓ cough, dyspnea, wheezing and nocturnal symptoms	Y	Mirsadraee et al. (2020)
		44.6 ± 18.5 years; 32 male, 20 female	Placebo group: 3 placebo tablets/day	↓ airway hyperresponsiveness		
		Propolis (*n* = 26)	Daily for 1 month	↑ Asthma Control Test scores		
		Placebo (*n* = 26)		↑ FEV1, FEV1/FVC, PEFR, FEF25-75		
				↓ FeNO↓ sputum eosinophils		
Atopic sensitization and eczema	Brazilian green propolis extract (Yamada Bee Farm Co., Ltd., Okayama, Japan)	Lactating women (>20 years old) having atopic symptoms and their offspring (2–8 months) having eczema	Propolis group: 3 propolis capsules/day (100 mg propolis extract/capsule)	↔ atopic sensitization (serum levels of total IgE and antigen-specific IgE for mites, egg white, cow’s milk, and wheat) at the first birthday of offspring	N	Igarashi et al. (2019)
		Propolis (*n* = 40)Placebo (*n* = 40)	Placebo group: 3 placebo capsules/day (propolis replaced with safflower oil)From the day of enrollment when their offspring were between 2 and 8 months of age until their first birthday	↔ non-specific symptoms (eczema) in mothers and their offspring		

Another study conducted by Tani et al. (2010) investigated the anti-allergic effects of Brazilian green propolis ethanol extract using peripheral leukocytes and peripheral blood mononuclear cells (PBMCs) isolated from patients who had a clinical history of pollinosis and were sensitized to Japanese cedar pollen, Cry j1/2 (Tani et al., 2010). Brazilian green propolis ethanol extract concentration-dependently inhibited CysLTs release in Cry j1-induced peripheral leukocytes with an IC_50_ value of 5.8 μg/ml. On the other hand, a significant inhibitory effect on histamine release was only observed at a high concentration of ethanol extract at 100 μg/ml. This implies that the bioactive compound(s) that inhibited histamine release may be present in a low quantity in the extract. In Cry j1-induced PBMCs, the propolis ethanol extract also non-significantly inhibited IL-5 and IL-13 production. As the Brazilian green propolis contains relatively higher amounts of artepillin C (20.7%), bacharrin (7.5%) and kaempferide (3.6%), these active constituents were believed to contribute to the inhibitory effect of the extract on CysLTs release. However, as suggested by the authors, it is crucial to determine whether the low abundance compound(s) will exert a more substantial effect than the high-abundance compounds.

Collectively, propolis has been shown to exert inhibitory effects against allergic inflammatory responses in different cellular models, including epithelial cells and immune cells such as peripheral leukocytes and PBMCs (Tani et al., 2010; Khosravi et al., 2018).

### Mast Cell Degranulation

Nakamura et al. (2010) conducted a study to compare the effects of water (WEP) and ethanol (EEP) extracts of propolis originated from China and Brazil on mast cell degranulation using a rat basophilic leukaemia cell line, RBL-2H3 (Nakamura et al., 2010). Anti-dinitrophenyl (DNP)-IgE sensitized-RBL-2H3 cells are commonly induced with DNP-bovine serum albumin (BSA) to simulate the mast cell degranulation process. It has been shown that WEP and EEP from China exerted more substantial inhibitory effects on β-hexosaminidase release in activated RBL-2H3 cells compared with WEP and EEP from Brazil. Regardless of the origin, EEP was reported to be more potent than WEP in suppressing mast cell degranulation, suggesting that the ethanol extract of propolis may contain more active constituents with anti-allergic properties than the water extract. Further analysis of China’s EEP revealed that chrysin and kaempferol are the primary active constituents contributing to the Chinese propolis’ anti-allergic activity. By contrast, chrysin and kaempferol were below detectable levels in Brazilian propolis, possibly explaining why Brazilian propolis showed weaker inhibitory effects on mast cell degranulation than Chinese propolis. Consistent with the finding reported by Nakamura et al. (2010), a few other studies by the same group of researchers (Shinmei et al., 2004, 2009, 2010) have also demonstrated the inhibitory effects of Brazilian green propolis ethanol extract on histamine release in rat peritoneal mast cells induced with compound 48/80, a commonly used mast cell activator. The results of propolis in allergic diseases such as asthma, allergic rhinitis and atopic dermatitis in which mast cell degranulation plays a crucial role are further discussed in subsequent sections.

### Basophil-Mediated Allergic Inflammation

Other than mast cells, propolis has also been shown to modulate the cellular responses of basophils. In a study by Kashiwakura et al. (2021), bone marrow-derived basophils (BMBs) induced with DNP_23_-human serum albumin (HSA) and treated with propolis at a concentration of 100 μg/ml showed a significantly reduced production of pro-inflammatory cytokines, IL-4, IL-6 and IL-13, by inhibiting the phosphorylation of Lyn, protein kinase B (Akt) and extracellular signal-regulated kinase (Erk) (Kashiwakura et al., 2021). Consistent with the *in vitro* finding, Kashiwakura et al. (2021) demonstrated that propolis treatment suppressed basophil-mediated skin and allergic intestinal inflammation *in vivo* using ovalbumin (OVA)-induced mouse models of chronic allergic inflammation (CAI) and food allergy, respectively. In the mouse model of CAI, propolis (0.3 mg) administered intragastrically reduced skin thickness and inflammation of ears. In mice with food allergy, oral propolis treatment (0.3 mg) also reduced diarrhoea occurrence, clinical scores, and serum levels of mouse mast cell protease-1 (mMCP-1). The gene expression of IL-4 and ectonucleotide pyrophosphatase/phosphodiesterase family member 3 (ENPP-3), a marker of activated basophil, in the jejuna was also reduced, suggesting that propolis treatment also inhibited basophil activation *in vivo*.

### Asthma

An *in vivo* study by Sy et al. (2006) evaluated the potential therapeutic effects of Taiwanese propolis water extract for asthma using OVA-induced asthmatic mice (Sy et al., 2006). Administration of propolis treatment (65 and 325 mg/kg) via tube feeding attenuated airway hyperresponsiveness and lung inflammation and reduced IL-5 levels in bronchoalveolar lavage fluid (BALF). However, the low dose (65 mg/kg) and high dose (325 mg/kg) propolis treatment had a distinct effect on the levels of OVA-specific IgE and IgG_1_ (Th2 response) and OVA-specific IgG_2α_ (Th1 response), where the low dose treatment decreased OVA-specific IgE and IgG_1_ with no effect on IgG_2α_. By contrast, the high dose treatment increased OVA-specific IgG_2α_ with no impact on OVA-specific IgE and IgG_1_. This indicates that the active constituents of the Taiwanese propolis water extract may consist of both Th1 and Th2 response regulators; however, further study is required to confirm that. Overall, Taiwanese propolis water extract may benefit asthma due to its modulatory effect on Th1 and Th2 responses.

Using a murine model of OVA-induced asthma, De Farias et al. (2014) demonstrated that propolis hydroalcoholic extract (PHE) reduced polymorphonuclear cell and total cell counts in BALF, peribronchovascular inflammation and epithelial desquamation in lungs as well as IFN-γ levels in serum, indicating that the propolis produced by *Scaptotrigona* aff. *postica* stingless bee in Brazil may have potential therapeutic effects for asthma by suppressing pulmonary inflammation (De Farias et al., 2014). Notably, when administered orally at 50 and 200 mg/kg to OVA-induced asthmatic mice, the PHE results were comparable to that of 1 mg/kg of intraperitoneal dexamethasone treatment. The authors claimed that propolis treatment might be safer than dexamethasone because immunosuppression was only observed on the mice receiving dexamethasone, but not propolis treatment.

Besides that, El-Aidy et al. (2015) conducted a study to investigate the anti-asthmatic effects of a few bee products, including propolis, honey, and royal jelly (El-Aidy et al., 2015). They demonstrated that propolis ethanol extract and water extract at a dose of 30 mg/kg reduced eosinophils and basophils in the blood and inflammatory cells in the peribronchial and perivascular regions in the lungs of conalbumin-induced asthmatic mice. However, assessments on the other pathological changes in asthma such as airway hyperresponsiveness, serum IgE levels, and Th1/Th2 cytokines were not carried out. Nonetheless, a comparison of both extracts’ chemical compositions may lead to identifying new anti-allergic compound(s).

A study by Piñeros et al. (2020) reported that a standardized Brazilian green propolis extract (EPP-AF®) was able to inhibit pulmonary inflammation and mucus production as well as to reduce IL-5 and eosinophils in BALF in OVA-induced asthmatic mice (Piñeros et al., 2020). EPP-AF® (150 mg/kg) administered via oral route was shown to increase the numbers of polymorphonuclear-myeloid derived suppressor cells (PMN-MDSC) and CD4^+^ Foxp3+ regulatory T cells, which have been shown to regulate Th2 responses in the lungs negatively. Therefore, the anti-asthmatic effects of EPP-AF® were likely related to its immunoregulatory activities.

### Allergic Rhinitis

An *in vivo* study by Shinmei et al. (2009) investigated the effects of Brazilian propolis granular obtained from Yamada Apiculture Center Inc., Okayama, Japan, on allergic rhinitis symptoms such as sneezing and nasal rubbing over 4 weeks (Shinmei et al., 2009). The study demonstrated that a single administration of propolis treatment (200, 500 and 1,000 mg/kg) via oral route had no effect on sneezing and nasal rubbing in OVA-sensitized mice induced with antigen. However, repeated administration of propolis resulted in a gradual improvement of symptoms in mice receiving 1,000 mg/kg of propolis treatment, where sneezing and nasal rubbing were significantly reduced from week two until week four. The effects of propolis were similar to a mast cell stabilizer, Tranilast, which also gradually reduced sneezing and nasal rubbing with significant inhibition observed in mice receiving oral treatment of Tranilast (300 mg/kg) starting from week three onwards. Neither single nor repeated administration of propolis treatment reduced serum IgE levels, suggesting that propolis has no inhibitory effect on IgE production. Propolis treatment also did not affect allergic rhinitis symptoms in non-sensitized mice induced with histamine, indicating that propolis did not block the action of histamine. On the other hand, Brazilian green propolis ethanol extract produced from the Brazilian propolis granular suppressed histamine release in mast cells induced with antigen or compound 48/80. Therefore, the beneficial effects of propolis on allergic rhinitis symptoms are likely to be due to the inhibition of histamine release by activated mast cells rather than blocking the activity of histamine.

Yasar et al. (2016) conducted a study to investigate the effects of poplar propolis produced by *Apis mellifera caucasica* honeybees in Turkey on allergic rhinitis (Yasar et al., 2016). The authors compared the anti-allergic activity of propolis ethanol extract administered via two different routes, which were oral and intranasal. The results showed that oral propolis was more effective than intranasal propolis in reducing inflammation, vascular proliferation, increase in goblet cells, and symptom scores (nasal irritation, sneezing, and amount of nasal secretion). Notably, the inhibitory effects of oral propolis at 200 mg/kg on inflammation and goblet cell counts in nasal mucosa as well as symptoms scores were more substantial than 50 µg intranasal mometasone furoate (a steroidal drug) and 10 mg/kg oral ketotifen (an antihistamine), suggesting the potential use of poplar propolis produced by *Apis mellifera caucasica* honeybees as an oral treatment for allergic rhinitis.

Another study by Shaha et al. (2018) evaluated the potential therapeutic effects of Brazilian green propolis ethanol extract in rats with 2, 4-diisocyanate (TDI)-induced allergic rhinitis (Shaha et al., 2018). In rats receiving Brazilian green propolis ethanol extract treatment (40 and 80 mg/kg) orally, sneezing, water rhinorrhea, swelling, and redness of nasal mucosa were attenuated. Gene expression levels of histamine H1 receptor (H1R) and pro-inflammatory cytokines such as IL-4, IL-5, and IL-9 in the nasal mucosa of TDI-induced rats receiving propolis treatment were also inhibited. This suggests that Brazilian green propolis could suppress inflammatory responses in the nasal mucosa other than providing symptomatic relief.

### Atopic Dermatitis

Brazilian green propolis has been shown to ameliorate itching in animal models of atopic dermatitis when administered orally (Shinmei et al., 2004) or applied topically (Shinmei et al., 2010). A study by Shinmei et al. (2004) assessed the effects of Brazilian green propolis granular on atopic dermatitis in rats induced with compound 48/80 or histamine. A single administration of propolis to the compound 48/80-induced rats via oral route at a dose of 1,000 mg/kg significantly suppressed scratching frequency and reduced skin vascular permeability. The inhibitory effects of propolis increased gradually when the treatment was continued daily for 4 weeks. Similar therapeutic effects of Brazilian green propolis for atopic dermatitis were demonstrated by another study by Shinmei et al. (2010), which assessed the efficacy of topical propolis to relieve itch in compound 48/80-induced rats. Interestingly, Brazilian green propolis ethanol extract applied at a dose of 3 mg per site on skin 60 min before the inducer compound 48/80 was injected intradermally suppressed scratching behaviours. The effect was prolonged until 60 min. The authors suggest that the prolonged effect of propolis against scratching might be due to its inhibition of the mast-cell dependent late-phase reaction. In contrast to oral propolis, which required an extremely high dose (1,000 mg/kg) to relieve itching, topical propolis was effective at a lower dose (3 mg/site). This suggests that topical propolis may be a more potent treatment for atopic dermatitis compared to oral propolis.

## Clinical Studies on the Effects of Propolis in Allergic Diseases

### Asthma

A comparative placebo-controlled clinical study by Khayyal et al. (2003) investigated the potential beneficial effects of a milk formula containing propolis aqueous extract as an adjuvant therapy for asthma (Khayyal et al., 2003) (Table 1B). In addition to oral theophylline as a regular medication, subjects with mild to moderate asthma drank milk prepared from propolis sachet or placebo sachet daily for 2 months. Significant reductions in the frequency and severity of nocturnal attacks and improvements in pulmonary ventilatory functions were observed among subjects in the propolis group. A decrease in serum levels of TNF-α, IL-6, IL-8, and intercellular adhesion molecule-1 (ICAM-1) and an increase in serum levels of IL-10 were observed. Serum levels of PGE_2_, PGF_2α_, and LTD_4_ were also reduced compared to baseline values. The authors reported that many subjects in the propolis group required fewer doses of their rescue medication, salbutamol, throughout the study period. These findings indicate that propolis aqueous extract as a nutritional supplement may be beneficial in asthma management.

Another clinical trial evaluating the effectiveness of propolis cera tablets to ameliorate asthma symptoms was conducted by Mirsadraee et al. (2020). Subjects with moderate persistent asthma were randomly assigned to receive three propolis cera tablets (each containing 75 mg propolis) or placebo tablets daily for a month. Propolis treatment attenuated asthma symptoms such as cough, dyspnea, wheezing, and nocturnal attacks. Airway hyperresponsiveness, pulmonary function, and asthma control also showed significant improvements. Furthermore, propolis treatment reduced fractional exhaled nitric oxide (FeNO) and sputum eosinophils in the propolis group. At the end of the trial, a reduced frequency of asthma attacks and emergency department visits was recorded among the subjects receiving propolis treatment. Overall, this study suggests the potential therapeutic effects of propolis for asthma.

### Atopic Sensitization and Eczema

A randomized, double-blind, placebo-controlled trial was conducted by Igarashi et al. (2019) to evaluate the effects of Brazilian green propolis extract given as a supplement to lactating women on atopic sensitization and eczema in their offspring (Igarashi et al., 2019). This study demonstrates that lactating women taking Brazilian green propolis did not affect the risk of their offspring in developing atopic sensitization on their first birthday, as indicated by similar serum levels of total IgE as well as antigen-specific IgE (mites, egg white, cow’s milk, and wheat) between the placebo and propolis groups. At the same time, the non-specific symptoms (eczema) neither improved nor worsened in the mothers and their offspring. While these findings seem to discourage the use of Brazilian green propolis extract for atopic conditions such as eczema, the authors suggested a few possibilities which could result in the lack of efficacy of propolis. Firstly, the sample size was small due to many dropouts (*n* = 26). Furthermore, we also noticed that no baseline values were taken for serum levels of total IgE and antigen-specific IgE. Comparison of serum IgE levels should also be made between the baseline and the first birthday of the same infant. Nevertheless, no serious adverse events were reported throughout the study period among the mothers and their offspring, except for one mother who had mild nausea that lasted temporarily, indicating that Brazilian green propolis is safe as a supplement for lactating women.

## Active Constituents of Propolis With Anti-Allergic Properties

**FIGURE 1:**
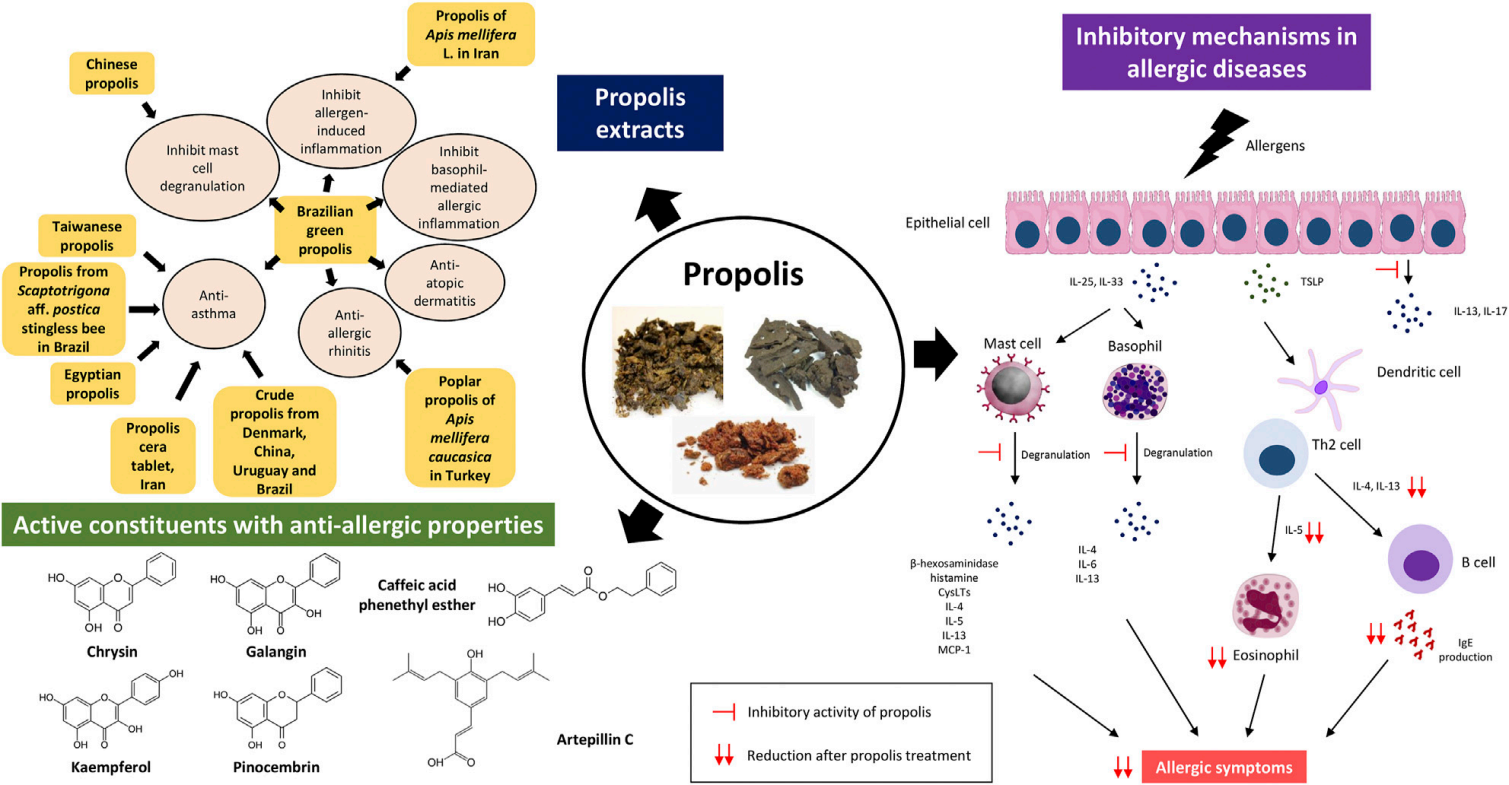
Different types of propolis extracts have shown anti-allergic effects against allergic inflammation, asthma, allergic rhinitis, and atopic dermatitis. Brazilian green propolis is the most promising remedy for allergic diseases as it has been demonstrated to inhibit several pathophysiological mechanisms in allergy. The anti-allergic properties of propolis may be partly attributed to the inhibitory activity of propolis on the activation of epithelial cells, mast cells, basophils, and eosinophils and the release of various allergic mediators. The active constituents of propolis with anti-allergic properties are flavonoids such as chrysin, kaempferol, galangin, and pinocembrin and cinnamic acid derivatives such as caffeic acid phenethyl esther (CAPE) and artepillin C. However, it should be noted that the chemical composition of propolis varies according to geographical locations, climates, botanical sources, and bee species. IL: Interleukin; TSLP: thymic stromal lymphopoietin; Th2: T helper 2; IgE: Immunoglobulin E; CysLTs: cysteinyl leukotrienes; MCP-1: monocyte chemoattractant protein-1.

Looking closely into the studies that reported anti-allergic activities of propolis, not all of them determined the chemical composition and active constituents of the propolis samples (Supplementary Table S1). As the chemical composition of propolis varies according to geographical locations, climates, botanical sources, and bee species (Katekhaye et al., 2019), it is difficult to relate the anti-allergic properties of propolis from different sources when the chemical composition is not known. However, several researchers have attributed the anti-allergic activities of propolis to the presence of flavonoids such as chrysin, kaempferol, galangin, and pinocembrin (Shinmei et al., 2009; Nakamura et al., 2010; Shinmei et al., 2010). These compounds have been demonstrated to have potential therapeutic effects for different allergic diseases *in vivo*, such as asthma (kaempferol, pinocembrin) (Gong et al., 2012; Gu et al., 2017), atopic dermatitis (chrysin) (Choi et al., 2017) as well as anaphylaxis (galangin) (Kim et al., 2013) (Figure 1).

Among the studies discussed here, Brazilian green propolis is the most common type of propolis that has been investigated for its anti-allergic activities. However, the potential active constituents contributing to these anti-allergic activities remain unknown, except for allergen-induced allergic inflammation *in vitro*. The active constituents were likely to be artepillin C, baccharin and kaempferide (Tani et al., 2010). Besides that, different sources of Brazilian propolis that have shown anti-allergic activities, such as the standardized Brazilian green propolis extract, EPP-AF® (Piñeros et al., 2020) and Brazilian green propolis from Yamada Bee Company Inc. (Shinmei et al., 2004; Shinmei et al., 2009; Shinmei et al., 2010; Ohkura et al., 2016; Shaha et al., 2018), contain compounds such as caffeic acid, coumaric acid, naringenin, kaempferide, and pinocembrin. The anti-allergic properties of these compounds remain to be fully explored.

As several studies have demonstrated the anti-allergic activities of Brazilian green propolis, it is surprising to note that the major constituent artepillin C has not been tested in experimental models of allergic diseases, except for an *in vitro* study by Tani et al. (2010) which evaluated the effects of artepillin C on allergen-induced allergic inflammation (Tani et al., 2010). Artepillin C is the most abundant compound of Brazilian green propolis that differentiates it from the other types of propolis from different regions (Huang et al., 2014). Future studies could be carried out to evaluate the anti-allergic potential of artepillin C. Besides that, caffeic acid phenethyl ester (CAPE) is a natural derivative of caffeic acid that has drawn many researchers’ attention due to its potent antioxidant, anti-inflammatory, and anticancer effects (Armutcu et al., 2015). CAPE has also been demonstrated to suppress OVA-induced active systemic anaphylaxis (Park et al., 2008) and asthma (Jung et al., 2008). However, Brazilian green propolis has been claimed to contain no CAPE (Berretta et al., 2017), including the standardized Brazilian green propolis that showed an anti-asthmatic effect (Hori et al., 2013; Piñeros et al., 2020). Although it is unclear whether some propolis extracts discussed here (Table 1) contain CAPE, CAPE was found in other sources of Chinese propolis (Chang et al., 2017), Turkish propolis (Özkök et al., 2021), and Egyptian propolis (Abd El Hady and Hegazi, 2002). Whether CAPE contributes to the reported anti-allergic activities of Chinese propolis (Nakamura et al., 2010), Turkish propolis (Yasar et al., 2016), and Egyptian propolis (El-Aidy et al., 2015) remains to be explored in future studies.

## Potential Therapeutic Use of Propolis in Allergic Diseases: Prospects and Challenges

Both preclinical and clinical studies have shown that propolis extracts have promising anti-allergic effects against allergic inflammation, asthma, allergic rhinitis, atopic dermatitis, and food allergy, which may be partly attributed to their inhibitory effects on the activation of effector cells such as mast cells and basophils. These findings indicate that propolis may be a potential remedy for allergic diseases. However, there is a lack of clinical trials, especially those evaluating the anti-allergic effects of the active constituents. Also, it should be noted that there are concerns about the increased prevalence of propolis allergy in some countries such as the United States (Shi et al., 2016), Singapore (Kwong and Lim, 2020), and Germany (Hausen, 2005). Notably, Hausen (2005) showed that CAPE is the primary contact allergen in propolis, followed by benzyl caffeate, 3-methyl-2-butenyl caffeate, and geranyl caffeate (Hausen, 2005). Besides that, it has been reported that up to 28.6% of beekeepers were sensitized to propolis, probably because of their higher exposure to propolis (Oosterhaven et al., 2019). Although it remains debatable whether propolis itself could serve as a sensitizing agent as the chemical composition of propolis varies (Valero Da Silva et al., 2019), it is reasonable to conclude that topical use of propolis may not be favourable for specific allergic individuals.

In this review, all three clinical studies discussed demonstrate that propolis is safe to be consumed as a supplement as no adverse events were reported among the asthmatic patients (Khayyal et al., 2003; Mirsadraee et al., 2020) or lactating women (Igarashi et al., 2019) who consumed propolis in the forms of capsule, tablet or milk product, except for one lactating mother who had mild and transient nausea (Igarashi et al., 2019). Furthermore, an *in vivo* study by Yasar et al. (2016) showed that oral propolis is more effective than topical (intranasal) propolis as a treatment for allergic rhinitis (Yasar et al., 2016). It is worthwhile to evaluate the potential of propolis as an adjunct therapy for allergic diseases, including allergic rhinitis and atopic dermatitis, in the clinical settings as Khayyal et al. (2003) demonstrated that the addition of propolis into the treatment regime for asthma with oral theophylline significantly improved the conditions of asthmatic patients (Khayyal et al., 2003). However, an allergy test may need to be conducted for individuals who want to use propolis as a CAM to treat allergic conditions and avoid undesirable side effects. Lastly, there are some significant challenges to the commercialization of propolis, including the lack of a standardization method for quality control of propolis from different sources due to its complex chemical composition, the difficulty in obtaining a quality source of propolis, and the declining bee populations (reviewed by Katekhaye et al., 2019). Although propolis is available in the market as a supplement, more studies are needed in the future to facilitate the development of propolis for prophylactic or therapeutic use in allergic diseases.

## Conclusion

In conclusion, propolis has promising potential to be further developed for prophylactic or therapeutic use in allergic diseases, such as asthma, allergic rhinitis, and atopic dermatitis, as its therapeutic benefits are well-supported by both preclinical and clinical studies. However, as the chemical composition of propolis varies according to geographical locations, climates, botanical sources, and bee species, future studies should relate the anti-allergic activity of propolis with its chemical composition particularly chrysin, kaempferol, galangin, pinocembrin, CAPE, and artepillin C to ensure the results reproducibility and to discover novel anti-allergic compounds. Clinical studies are also required to evaluate the effects of these active constituents on allergic diseases. Besides that, it is worthwhile to assess the efficacy of propolis as a supplement or an adjunct therapy to treat allergic rhinitis and atopic dermatitis in clinical settings as such an approach has been proven to be safe and effective for asthmatic patients.
