# Supplementary material for: Anti-Allergic Properties of Propolis: Evidence From Preclinical and Clinical Studies
Source: Front Pharmacol. 2022 Jan 21;12:785371. doi: 10.3389/fphar.2021.785371 (PMC8816323; doi:10.3389/fphar.2021.785371)
Supplement: Supplementary file 1 [file Table1.DOCX]

Supplementary Material

# Supplementary Figures and Tables

## Supplementary Tables

**Supplementary Table 1.** Chemical composition and active constituents of different types of propolis with anti-allergic properties.

| Type of allergic disease | Type of propolis | Extraction method | Chemical composition | Active constituents | Reference |
| --- | --- | --- | --- | --- | --- |
| Allergen-induced allergic inflammation | Propolis ethanol extract (propolis collected from hives of *Apis mellifera* L. bees in Alikhani Farm of southern Iran) | Extracted with 95% ethanol, filtered and evaporated at 25℃ | Benzoic acid, p-Methoxy-Cinnamic acid, 3,4-Dimethoxy-Cinnamic acid, Ferulic acid, Isoferulic acid, 4-Methoxy-Cinnamic acid, Caffeic acid, Succinic acid, Palmitic acid, Octadecadienoic acid, D-Fructose, Glucofuranose, Talose, Treitol Polycyclic aromatic hydrocarbons (PAH), 7,14-Dihydro-dibenz[a,j]anthracene, (R)-(1)-2-Amino-20-(isopropylamino)-1,10-binaphthyl, 1,3,6,7,8-Pentamethyl-5,6,7,8-tetrahydro-2,4(1H,3H)-pteridindione, syn-Phenanthro[9,10:10,20][2.2]metacyclophan-1-ene, 2-Imino-5-phenyl-1,2-dihydro-3H-1,3,4-benzotriazepine Phenolics, 3-Methyl-3-butenyl isoferulate, 2,6-Di-t-butyl-4-nitro-phenol (DBNP), 5-(Hydroxymethyl)resorcinol, Methylindan, Phoratoxon sulfone, Benzazepin derivatives, Pentachlorobenzenamine, 2,5-Bis(4-methylphenyl)-1,4-dithiin, Methylthiouracil, p-Toluidine, Pinostrobin chalcone, 4-Methyl-6,7-dimethoxy-2H-1,3-benzothiazine, Chalcone, 2,4-Bis(methylthio)-5,6-dihydro-8-methoxybenz[f]isoquinoline, 9,10-Bis(7-deuterobicyclo[4.1.0]hept-7-yl)-9,10-dihydro-anthracene, 2-1-(methylethyl)-8-oxo-1,2-dihydrofurano[2,3-H] 2H-chromene, Buxozine-C, Salbutamol, Delta 1-tetrahydrocannabinolic acid, Guaifenesin | Unknown | (Khosravi et al., 2018) |
|  | Brazilian green propolis ethanol extract (Apiários Floresta, Minas Gerais, Brazil) | Extracted with ethanol, filtered and evaporated until the solid content reached 55% | Artepillin C (20.7%), Caffeic acid (0.1%), Drupanin (1.9%), Baccharin (7.5%), Culifolin (0.3%), Kaempferol (0.2%), Kaempferide (3.6%), Pinocembrin (3.1%) | Artepillin C, Baccharin, Kaempferide | (Tani et al., 2010) |
| Mast cell degranulation | Water extract of propolis from China | Extracted with ultrapure water | Not reported | Unknown | (Nakamura et al., 2010) |
|  | Ethanol extract of propolis from China | Extracted with 95% ethanol | Chrysin, Galangin, Kaempferol, 3-O-methylkaempferol | Chrysin, Kaempferol |  |
|  | Water extract of propolis from Brazil | Extracted with ultrapure water | Not reported | Unknown |  |
|  | Ethanol extract of propolis from Brazil | Extracted with 95% ethanol | Not reported, Chrysin and Kaempferol below detectable level | Unknown |  |
|  | Brazilian propolis ethanol extract (Yamada Apiculture Center, Okayama, Japan) | Propolis extract as solid content in ethanol solution at 55% (w/v) | Chlorogenic acid (1.2 mg/g), Coumaric acid (12 mg/g), Caffeic acid (1.4 mg/g), Drupanin (14 mg/g), Artepillin C (95 mg/g), Baccharin (35 mg/g), Chrysin (0.029 mg/g), Naringenin (0.019 mg/g), Pinocembrin (0.37 mg/g), Kaempferide (17 mg/g), Kaempferol (1.1 mg/g  (Adapted from Ohkura et al., 2016) | Unknown | (Shinmei et al., 2004, 2009, 2010) |
| Basophil-mediated allergic inflammation | Propolis powder (Yamada Bee Company, Inc., Okayama, Japan) | NA | Standardized to contain 8% artepillin C | Unknown | (Kashiwakura et al., 2021) |
| Asthma | Propolis water extract powder (Uni-President Enterprises Corp., Taiwan) | NA | Not reported | Unknown | (Sy et al., 2006) |
|  | Propolis hydroalcoholic extract produced by *Scaptotrigona* aff. *postica* stingless bee (Barra do Corda, Maranhão, Brazil) | Macerated in 70% ethanol (24hr), filtered and concentrated at 40℃ in a rotary evaporator under low pressure | Flavonoids (0.55 ± 0.07%), phenolic acid (11.40 ± 0.73%), and total phenol contents (11.95 ± 0.80%) | Unknown | (De Farias et al., 2014) |
|  | Crude propolis ethanol extract (Agricultural Research Centre Station, Manzala, Dakahlyia, Egypt) | Extracted with 95% ethyl alcohol, filtered and dried at 60℃ | Not reported | Unknown | (El-Aidy et al., 2015) |
|  | Crude propolis aqueous extract (Agricultural Research Centre Station, Manzala, Dakahlyia, Egypt) | Extracted with water, filtered and dried at 60℃ | Not reported | Unknown |  |
|  | Standardized Brazilian green propolis extract (EPP-AF^®^ extract) (Apis Flora Co., Ribeirão Preto, São Paulo, Brazil) | Hydroalcoholic extraction using maceration followed by a turbo extraction process | Caffeic acid (1.90 ± 0.014 mg/g), p-coumaric acid (10.016 ± 0.028 mg/g), 3,5-dicafeoyl quinic (3,5-DCQ) (14.293 ± 0.08 1mg/g), 4,5-DCQ (18.364 ± 0.164 mg/g), aromadendrin-4-O-methyl-ether (2.519 ± 0.023 mg/g), drupanin (17.343 ± 0.072 mg/g), artepillin C (50.299 ± 1.039 mg/g) and baccharin (8.459 ± 0.281 mg/g), Total flavonoid content as quercetin (41.449 ± 0.425 mg/g) and total polyphenol as gallic acid (150.37 ± 0.78 mg/g) | Unknown | (Piñeros et al., 2020) |
|  | Propolis aqueous extract (Propharma, Stenlose, Denmark) prepared from crude propolis collected from Denmark, China, Uruguay and Brazil | Aqueous decoction of crude propolis, aqueous extract concentrated, spray-dried under high pressure and incorporated into milk formula | Standardized to contain no less than 0.05% of organic aromatic acids, mainly caffeic, ferulic, iso-ferulic, cinnamic and 3,4-dimethoxy-cinnamic acids, in addition, to trace amounts of various flavonoids | Unknown | (Khayyal et al., 2003) |
|  | Propolis cera tablet (Soren Tech Toos Company, Mashhad, Iran) | NA | Not reported | Unknown | (Mirsadraee et al., 2020) |
| Allergic rhinitis | Brazilian propolis granular (Yamada Apiculture Center, Inc., Okayama, Japan) | NA | Not reported | Unknown | (Shinmei et al., 2009) |
|  | Propolis ethanol extract (poplar propolis collected from honeybee colonies of *Apis mellifera caucasica*, Kayseri, Central  Anatolia, Turkey) | Extracted with 70% ethanol, filtered and evaporated using a vacuum evaporator | 4,5 Dimethoxy-(2-propenyl) 2-phenol, Pinocembrin, 5-Methoxy-3,7-dihydroxyflavanone, 5-Hydroxy-7-methoxy flavone (tectochrysine), 7-Dihydroxy-3-methoxyisoflavone, Chrysin, Galangin, 4,5-Dihydroxy-7-methoxyflavanone, Decanoic acid, 4-Pentenoic acid, Cinnamic acid, 3-Hydroxy-4methoxycinnamic acid, 2-propenoic acid, 3,4-Dimethoxycinnamic acid, n-Hexadecanoic acid, Coumaric acid, m-Hydroxycinnamic acid, Octadecanoic acid, 1,2-Benzenedicarboxylic acid, Nerolidol, 2-Propen-1-ol, Benzenemethanol, Chrysophanol, 5-3,3-Dimethyl-cyclohexanone, Ethanone, 3-Methoxy acetophenone, 4H-pyrazolopyrimidin-4-one, Oxacyclododeca-6,9-dien-2-one, 2-Nonadecanone, Gamma-eudesmol, Beta-eudesmol, Alpha-eudesmol, Alpha-bisabolol, 1-Pentanone, 2-Propen-1-one, 2,3-Diphenylcyclopetanone, Benzofuran, 6,8 Tetradecadiene, 1-Buta-1,3-diene, 3,6 Dimethoxy-2-ethylbenzaldehyde, 4-Pyrimidinamine, Heptane-2-propanoate, 3-Cyano-5,6-dimethoxy-2-methylthio-1-indole-1-phenylindole  (Adapted from Mişe Yonar et al., 2014) | Unknown | (Yasar et al., 2016) |
|  | Brazilian green propolis ethanol extract (Yamada Bee Company, Inc., Okayama, Japan) | Propolis extract as solid content in ethanol solution at 55% (w/v) | 8% artepillin C | Unknown | (Shaha et al., 2018) |
| Atopic dermatitis | Brazilian propolis granular (Yamada Apiculture Center,  Okayama, Japan) | NA | Not reported | Unknown | (Shinmei et al., 2004) |
|  | Brazilian propolis ethanol extract (Yamada Apiculture Center,  Okayama, Japan) | Propolis extract as solid content in ethanol solution at 55% (w/v) | Chlorogenic acid (1.2 mg/g), Coumaric acid (12 mg/g), Caffeic acid (1.4 mg/g), Drupanin (14 mg/g), Artepillin C (95 mg/g), Baccharin (35 mg/g), Chrysin (0.029 mg/g), Naringenin (0.019 mg/g), Pinocembrin (0.37 mg/g), Kaempferide (17 mg/g), Kaempferol (1.1 mg/g)  (Adapted from Ohkura et al., 2016) | Unknown | (Shinmei et al., 2010) |
